# Frequency-Dependent Whole-Brain Reconfiguration Following Left DLPFC rTMS in Older Adults: A 106-Channel fNIRS Study

**DOI:** 10.3390/s26072182

**Published:** 2026-04-01

**Authors:** Yingpeng Wang, Yingqi Li, Hujun Wang, Congxiao Wang, Anda Xiu, Jingxuan Wang, Shaoting Zhang, Chenye Qiao, Tingyu Jiang, Shuyan Qie

**Affiliations:** 1 Department of Rehabilitation, Beijing Rehabilitation Hospital, Capital Medical University, Beijing 100144, China; ypwang@ccmu.edu.cn (Y.W.); lyq9874@163.com (Y.L.); hujunwang@ccmu.edu.cn (H.W.); wangcongxiao66@163.com (C.W.); jiangty0929@163.com (T.J.); 2 School of Beijing Rehabilitation, Capital Medical University, Beijing 100144, China; xad999@mail.ccmu.edu.cn (A.X.); wangjingxuanella@mail.ccmu.edu.cn (J.W.); m13363206851@163.com (S.Z.); qcy0424@mail.ccmu.edu.cn (C.Q.)

**Keywords:** non-invasive neuromodulation, rTMS, DLPFC, resting-state functional connectivity, functional near-infrared spectroscopy, healthy aging

## Abstract

**Objective:** The classic excitation/inhibition dichotomy may be insufficient to describe rTMS mechanisms in the aging brain. This study investigated immediate whole-brain resting-state functional connectivity effects of 10 Hz (high-frequency) and 1 Hz (low-frequency) rTMS over the left dorsolateral prefrontal cortex (DLPFC) in healthy older adults. **Methods:** Thirty healthy older adults (aged 60–75 years) participated in a randomized, single-blind, crossover study, and underwent 20-min 10 Hz and 1 Hz rTMS in separate visits. A 106-channel fNIRS system was used to record resting-state activity before and immediately after each intervention. Functional connectivity was analyzed at the channel, region-of-interest (ROI) and network summary levels, including graph-theoretic metrics and distance-stratified connectivity summaries. **Results:** At the network summary level, 10 Hz stimulation was associated with relatively more positive changes in global topology and spatially distributed connectivity summaries, whereas 1 Hz stimulation showed the opposite overall trend. In the graph-theoretic analyses, stimulation frequency × time interaction effects were observed for global efficiency, local efficiency, clustering coefficient, and mean node strength. At the edge level, only a small number of effects survived FDR correction, and the broader connection-wise patterns were therefore interpreted as exploratory. Uncorrected analyses suggested widespread enhancement after 10 Hz stimulation and widespread reduction after 1 Hz stimulation, together with localized paradoxical effects, including selective decreases after 10 Hz and selective increases after 1 Hz (e.g., bilateral primary motor cortex connectivity). **Conclusions:** These findings suggest that 10 Hz and 1 Hz rTMS over the left DLPFC are associated with different patterns of immediate whole-brain network reconfiguration in healthy older adults. The presence of localized paradoxical effects further suggests that rTMS responses in the aging brain may involve more complex forms of reorganization than a simple excitatory/inhibitory dichotomy would predict. **Significance:** The present study provides preliminary support for a network-level perspective on neuromodulation in older adults and highlights the value of whole-brain fNIRS for characterizing distributed responses to rTMS. Larger, sham-controlled, behavior-linked, and longitudinal studies are needed to determine the robustness and functional significance of these effects.

## 1. Introduction

Population aging constitutes a formidable global public health challenge, bringing with it an increased prevalence of age-related cognitive decline. This demographic shift has catalyzed a paradigm change in neuroscience and clinical practice, moving from a focus on treating manifest disease to an emphasis on preventative neurology and the maintenance of cognitive resilience throughout the lifespan. National health strategies, such as China’s “Healthy China 2030” initiative, increasingly prioritize proactive health and functional maintenance, underscoring the urgent need for interventions that can preserve brain health in older adults. The normal aging process is accompanied by a complex reorganization of brain structure and function, including gray matter atrophy, reduced white matter integrity, and altered neural network connectivity patterns [1,2]. These changes are not uniform, exhibiting specific spatio-temporal patterns, with pronounced decline in prefrontal and temporal regions and relative preservation of primary sensorimotor areas [3]. Importantly, the aging brain does not passively succumb to these structural insults but actively compensates through functional reorganization to maintain cognitive performance. Theoretical frameworks such as the hemispheric asymmetry reduction in older adults (HAROLD) model and the posterior-to-anterior shift in aging (PASA) model describe these unique compensatory strategies [4,5,6]. Therefore, developing interventions that can effectively modulate neuroplasticity in the aging brain is not only a scientific imperative for combating cognitive decline but also aligns with national health policies emphasizing prevention and functional maintenance.

Repetitive transcranial magnetic stimulation (rTMS), as a non-invasive neuromodulation technique, offers tremendous potential for improving cognitive function in older adults through modulation of neuronal excitability and synaptic plasticity [3,7]. Extensive research demonstrates that rTMS can improve multiple cognitive domains in healthy older adults and patients with mild cognitive impairment, including memory, attention, and executive function [8,9,10,11,12]. Traditional rTMS effect theory follows a frequency-dependent dichotomy: high-frequency (≥5 Hz) stimulation enhances cortical excitability through long-term potentiation (LTP)-like mechanisms, while low-frequency (≤1 Hz) stimulation produces inhibitory effects through long-term depression (LTD)-like mechanisms [13,14,15,16]. While this framework provides guidance for clinical applications, its oversimplification is increasingly apparent. Multiple studies have reported “paradoxical” effects inconsistent with expectations, such as low-frequency stimulation producing excitatory effects under specific circumstances, suggesting that the true effects of rTMS are far more complex than the dichotomous model and may be jointly modulated by multiple factors including brain state, network properties, and homeostatic plasticity [14,17,18,19].

From a network neuroscience perspective, cognitive function does not depend on the isolated operation of single brain regions but rather emerges from the coordinated activity of distributed brain networks [20]. Functional connectivity, the temporal correlation of neural activity between different brain regions, represents a core metric for evaluating brain network organization and function. Older adults’ brain networks exhibit unique reorganization patterns: on one hand, within-network connections weaken and modularity decreases, manifesting as functional “dedifferentiation”; on the other hand, between-network connections increase, particularly enhanced interhemispheric connections, potentially reflecting activation of compensatory mechanisms [3,21]. Understanding how rTMS modulates these age-specific brain network patterns is crucial for optimizing stimulation protocols and achieving precision interventions.

Functional near-infrared spectroscopy (fNIRS) is an ideal neuroimaging modality for investigating the network-level effects of rTMS, particularly in older populations. Its immunity to the electromagnetic artifacts generated by the TMS coil, high tolerance for motion [22], and portability make it superior to concurrent TMS-fMRI or TMS-EEG for this purpose. While previous studies have combined rTMS and fNIRS, they have often been limited by low-channel counts, restricting analysis to local regions and precluding a whole-brain network assessment [22,23].

Therefore, how different frequency rTMS systematically reshapes whole-brain functional connectivity networks in older adults, and whether this reshaping follows simple excitation/inhibition rules, remains an unresolved critical scientific question. Addressing these questions will help deepen our understanding of rTMS mechanisms and provide scientific basis for developing individualized, precision neuromodulation protocols. This study aims to utilize 106-channel whole-brain coverage fNIRS technology to directly compare the immediate whole-brain network modulation effects of 10 Hz high-frequency versus 1 Hz low-frequency rTMS in healthy older adults. We hypothesize that: (1) 10 Hz stimulation will enhance whole-brain functional connectivity and promote network integration; (2) 1 Hz stimulation will reduce functional connectivity, leading to network segregation; and (3) both frequencies may elicit localized paradoxical connectivity changes, reflecting the brain’s complex homeostatic plasticity and compensatory mechanisms. Through analyzing network changes at whole-brain channel, region-of-interest (ROI), and network summary levels, this study aimed to characterize the immediate whole-brain effects of frequency-dependent rTMS in older adults, providing a basis for future mechanistic and translational studies.

## 2. Methods

### 2.1. Participants

Thirty right-handed, healthy older adults (13 males, 17 females; mean age 66.4 ± 4.2 years; mean height 163.67 ± 7.60 cm; mean weight 68.43 ± 11.43 kg) were recruited from the local community. Inclusion criteria were: age between 60 and 75 years, right-handedness as assessed by the Edinburgh Handedness Inventory, and a Mini-Mental State Examination (MMSE) score of ≥27. Exclusion criteria included any history of neurological or psychiatric disorders, contraindications to rTMS (e.g., history of epilepsy, presence of metallic implants), diagnosis of severe cardiovascular disease, or current use of medications known to affect cortical excitability. The study protocol was approved by the Ethics Committee of Beijing Rehabilitation Hospital, Capital Medical University (Approval No. 2022bkky-061-001). All participants provided written informed consent in accordance with the Declaration of Helsinki.

### 2.2. Experimental Design and Procedure

The study employed a single-blind, randomized, counterbalanced crossover design. Participants were not informed of the stimulation frequency administered in each session. Each participant attended two experimental sessions, one for 10 Hz rTMS and one for 1 Hz rTMS, separated by a one-week washout period to minimize potential carryover effects. The order of the stimulation conditions was randomized and balanced across participants. Each session followed an identical procedure: (1) a 5-min baseline resting-state fNIRS recording, during which participants were instructed to sit quietly with their eyes open and fixated on a central crosshair; (2) a 20-min rTMS intervention; and (3) an immediate 5-min post-stimulation resting-state fNIRS recording under the same conditions as the baseline.

### 2.3. rTMS Protocol

Stimulation was delivered using a YRD CCY-II magnetic stimulator and a 70 mm figure-of-eight coil (Wuhan Yiruide Medical Equipment New Technology Co., Ltd., Wuhan, China). The stimulation target was the left dorsolateral prefrontal cortex (DLPFC), localized at the F3 position of the International 10–20 EEG system as a practical scalp-based approximation, and no MRI-guided neuronavigation was used. The resting motor threshold (RMT) was determined for each participant as the minimum single-pulse intensity required to elicit a visible muscle twitch in the contralateral abductor pollicis brevis muscle in at least 5 out of 10 trials. The stimulation intensity for the main experiment was set to 90% of the individual’s RMT to ensure participant tolerance while robustly activating the cortical target [22]. During stimulation, the coil was held tangential to the scalp with the handle angled 45° posteriorly from the midline.

Two stimulation protocols with a matched total pulse count were administered:

High-Frequency (10 Hz) Protocol: Stimulation was delivered at 10 Hz in 1-s trains, with a 9-s inter-train interval. A total of 120 trains were administered over 20 min, delivering 1200 pulses.

Low-Frequency (1 Hz) Protocol: Stimulation was delivered continuously at 1 Hz for 20 min, also delivering 1200 pulses.

### 2.4. fNIRS Data Acquisition

**Figure 1:**
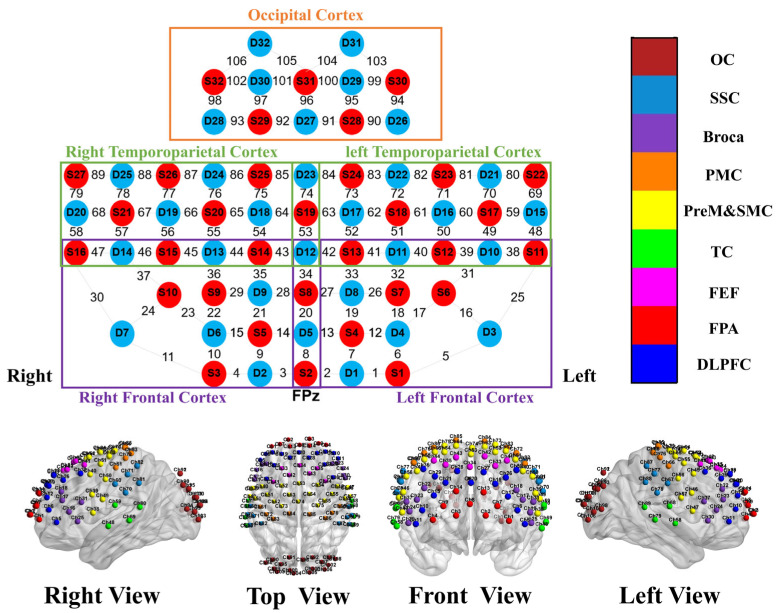
Distribution of fNIRS source-detector channels and corresponding whole-brain region parcellation. DLPFC: dorsolateral prefrontal cortex; FPA: frontopolar area; FEF: frontal eye field; TC: temporal cortex; PreM&SMC: premotor/supplementary motor cortex; PMC: primary motor cortex; Broca: Broca’s area; SSC: somatosensory cortex; OC: occipital cortex.

fNIRS data were acquired using a 106-channel system (Model BS-7000L, Wuhan Znion Technology Co., Ltd., Wuhan, China) with a sampling rate of 20 Hz. The system used two wavelengths of near-infrared light, 690 nm and 830 nm, to measure changes in hemoglobin concentrations. The probe set consisted of 32 light sources and 32 detectors arranged with a fixed inter-optode distance of 30 mm, providing coverage of bilateral frontal, parietal, temporal, and occipital cortices (Figure 1). Based on anatomical landmarks and Brodmann areas, 18 bilateral regions of interest (ROIs) were defined for analysis, including bilateral DLPFC, frontopolar area (FPA), frontal eye field (FEF), temporal cortex (TC), primary motor cortex (PMC), premotor/supplementary motor cortex (PreM&SMC), Broca’s area, somatosensory cortex (SSC), and occipital cortex (OC), as shown in Figure 1.

### 2.5. fNIRS Data Preprocessing

Data preprocessing and connectivity computation were performed by an analyst blinded to stimulation condition using the same pre-specified pipeline across sessions. fNIRS data were preprocessed using the Homer2 toolbox [24] implemented in MATLAB (R2021a, The MathWorks, Inc., Natick, MA, USA). The preprocessing pipeline consisted of the following steps: (1) Raw light intensity signals were converted to changes in optical density. (2) Motion artifacts were identified and corrected using a combination of spline interpolation and wavelet filtering [25]. (3) The data were then band-pass filtered between 0.01 Hz and 0.1 Hz to isolate low-frequency spontaneous fluctuations characteristic of resting-state activity and to remove physiological noise. (4) The modified Beer–Lambert Law was applied to convert the filtered optical density data into concentration changes of oxygenated hemoglobin (HbO) and deoxygenated hemoglobin (HbR). All subsequent functional connectivity analyses were performed on the HbO signal, which is considered to have a superior signal-to-noise ratio and a stronger correlation with underlying neural activity.

Signal quality control was performed separately for each participant and session using the coefficient of variation (CV). Channels with an absolute CV greater than 15% were classified as poor quality, excluded for that specific session, and masked prior to correlation computation. Across all sessions, the number of excluded channels was 9.3 ± 7.5 (median = 9) out of 106 channels.

### 2.6. Functional Connectivity Analysis

Functional connectivity (FC) was defined as the temporal correlation between brain regions. For each participant and condition, we constructed two types of connectivity matrices. First, a 100 × 100 whole-brain channel-level matrix was created by calculating the Pearson correlation coefficient between the HbO time series of all valid channel pairs (4950 unique connections). Six anatomically midline channels were excluded a priori because they could not be unambiguously assigned to either hemisphere, yielding a nominal 100-channel bilateral matrix for channel-level analyses. Second, an 18 × 18 ROI-level matrix was created by first averaging the HbO time series of all channels within each ROI and then calculating the Pearson correlation coefficients between all ROI pairs (153 unique connections). To satisfy the normality assumption for parametric statistical tests, all correlation coefficients were converted to z-scores using Fisher’s r-to-z transformation.

In addition to edge-wise FC analyses, we derived network-level and node-level topology measures from the weighted ROI-level connectivity matrices. Network-level metrics included global efficiency, local efficiency, clustering coefficient, small-worldness, modularity, participation coefficient, and mean node strength. Small-worldness (σ) was computed as (C/Crand)/(L/Lrand), where Crand and Lrand were estimated from degree- and density-matched random networks (100 realizations). Node-wise summaries included nodal strength and nodal efficiency across the 18 ROIs. For additional lower-dimensional channel-level summaries, all valid channel-to-channel connections were further classified into four spatial categories: intra-ROI, intrahemispheric, interhemispheric homologous, and interhemispheric heterologous connections.

### 2.7. Statistical Analysis

All statistical analyses were performed using MATLAB R2021a. Analyses were conducted at three levels: (1) edge-wise channel- and ROI-level connectivity comparisons; (2) network-level graph-theoretic summaries; and (3) lower-dimensional spatial summaries, including node-wise topology maps and distance-stratified channel-level change summaries.

**Figure 2:**
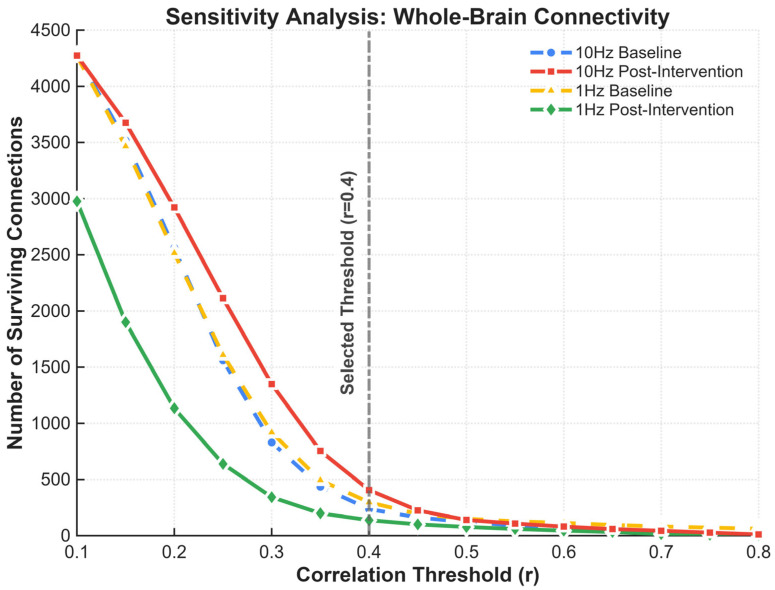
Sensitivity analysis of the descriptive connectivity threshold. The number of surviving channel-level connections is plotted across correlation thresholds ranging from r = 0.1 to r = 0.8 for each condition. The descriptive threshold of r = 0.4 was selected near the elbow region of the density curves to balance network contiguity and noise suppression. This threshold was used solely for visualization and did not enter inferential statistical analyses.

At the edge level, one-sample t-tests were first used to assess whether FC in each condition was significantly different from zero. To visualize and focus on physiologically meaningful connections while reducing noise, we applied a descriptive display threshold of r ≥ 0.4, such that only moderate-to-strong connections were shown in the connectivity graphs. This threshold was used solely for visualization and did not enter any inferential statistical tests. To evaluate the robustness of this descriptive threshold, we additionally performed a sensitivity analysis across correlation thresholds ranging from 0.1 to 0.8. Based on the resulting density curves, r = 0.4 was selected near the elbow region to balance network contiguity with noise suppression for visualization purposes only (Figure 2).

To assess stimulation effects, paired t-tests were conducted to compare: (1) FC changes from pre- to post-stimulation for each frequency; (2) baseline FC differences between the two sessions; and (3) post-stimulation FC differences between the two frequencies. In addition, change scores (Δ = post − pre) were calculated for each participant and condition, and paired comparisons of Δ10 Hz versus Δ1 Hz were used to assess whether between-session differences reflected distinct within-session trajectories. All edge-wise comparisons were corrected for multiple comparisons using the False Discovery Rate (FDR) at q < 0.05. Given the exploratory nature of this study and its sample size, we also report uncorrected results (*p* < 0.05) and Cohen’s d effect sizes to describe the overall patterns and magnitude of rTMS-induced effects. For visualizing whole-brain connectivity changes, we used a threshold of d ≥ 0.6 to highlight connections with medium-to-large effect sizes, consistent with Cohen’s medium effect size standard (d = 0.5–0.8).

At the network level, graph-theoretic metrics were analyzed using a 2 (Condition: 10 Hz vs. 1 Hz) × 2 (Time: Pre vs. Post) repeated-measures analysis of variance (rmANOVA). These analyses were used to quantify frequency-dependent changes in global network topology. At the node level, nodal strength and nodal efficiency were examined as descriptive spatial summaries of topology-level effects across the 18 ROIs. To obtain lower-dimensional summaries of channel-level effects by spatial scale, participant-level mean change scores (Δz = post − pre) were first computed within each spatial category for each participant and condition. Paired comparisons of Δ10 Hz versus Δ1 Hz were then performed across participants, and Holm correction was applied across the four spatial categories.

## 3. Results

We first describe the overall whole-brain connectivity architecture under each condition and then examine stimulation-related differences using formal statistical comparisons across channel-level and ROI-level networks.

### 3.1. Channel-Level Functional Connectivity Networks

#### 3.1.1. Pre- vs. Post-10 Hz rTMS

**Figure 3:**
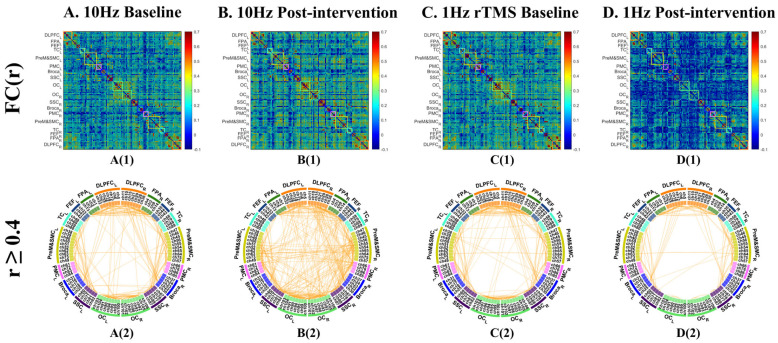
Channel-level functional connectivity architecture before and after 10 Hz and 1 Hz rTMS. (**A**) Baseline network before 10 Hz stimulation; (**B**) post-10 Hz network; (**C**) baseline network before 1 Hz stimulation; (**D**) post-1 Hz network. For each condition, panel (**1**) shows the full channel-by-channel functional connectivity (FC) matrix, and panel (**2**) shows descriptively robust connections (r ≥ 0.4). The r ≥ 0.4 threshold was used for visualization only and did not enter inferential statistical tests.

At baseline, the channel-level functional network showed a modular architecture with 237 robust connections (r ≥ 0.4), concentrated primarily within local cortical regions. Interhemispheric links were relatively sparse and were mainly observed between bilateral DLPFC, FPA, and OC, as shown in Figure 3(A1,A2).

Following 10 Hz rTMS, the descriptive network topology became denser, with the number of robust connections increasing to 406, representing a 71.3% increase from baseline (Figure 3(B1,B2)). Newly visible connections included both strengthened local links and more long-range and interhemispheric connections, suggesting a shift toward a more integrated global architecture.

**Figure 4:**
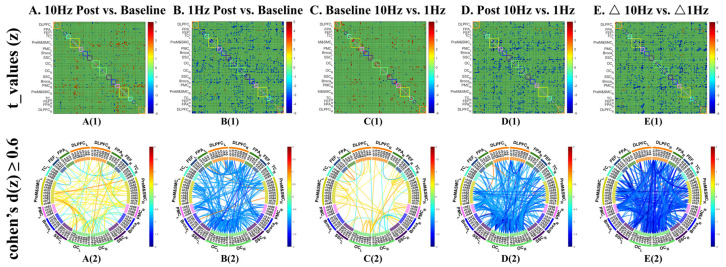
Channel-wise functional connectivity comparisons across rTMS frequency (10 Hz and 1 Hz) and time conditions (baseline and post-intervention). (**A**) Post-10 Hz versus baseline; (**B**) post-1 Hz versus baseline; (**C**) baseline 10 Hz versus baseline 1 Hz; (**D**) post-10 Hz versus post-1 Hz; (**E**) Δ10 Hz versus Δ1 Hz, where Δ = post − pre. Panel (**1**) shows *t*-values for channel-wise FC differences corresponding to uncorrected *p* < 0.05. Panel (**2**) shows channel pairs meeting the exploratory visualization criterion of uncorrected *p* < 0.05 and Cohen’s d ≥ 0.6. Edge-wise comparisons that did not survive false discovery rate (FDR) correction are presented as exploratory.

In the confirmatory edge-wise analysis, no individual channel-to-channel connections showed a significant pre-post change after FDR correction (q < 0.05). In the uncorrected analyses, however, the overall pattern suggested widespread strengthening (Figure 4(A1,A2)). A total of 83 channel pairs met the exploratory visualization criterion (uncorrected *p* < 0.05 and Cohen’s d ≥ 0.6), of which 61 (73.5%) showed increased connectivity. These increases were distributed across both hemispheres and were particularly evident in motor-related networks involving bilateral PMC and PreM&SMC. The minority of weakened connections were primarily observed between OC and Broca-related channels and among frontoparietal channels (uncorrected *p* < 0.05 and Cohen’s d ≥ 0.6).

#### 3.1.2. Pre- vs. Post-1 Hz rTMS

The baseline network prior to 1 Hz stimulation was broadly similar to that observed before 10 Hz stimulation, with 274 robust connections and a comparable modular structure (Figure 3(C1,C2)). In contrast to the 10 Hz condition, the descriptive network after 1 Hz rTMS appeared markedly sparser, with robust connections decreasing to 138, corresponding to a 49.6% reduction. This reduction was not confined to the stimulation target but extended across both intra- and inter-hemispheric links (Figure 3(D1,D2)).

In the confirmatory analysis, two channel pairs showed significant decreases after FDR correction (q < 0.05): one within the stimulated left DLPFC and one within the left OC. Uncorrected analyses further suggested a widespread predominance of weakened connectivity. A total of 156 channel pairs met the exploratory visualization criterion (uncorrected *p* < 0.05 and Cohen’s d ≥ 0.6), of which 153 (98.1%) represented decreases in connectivity. Only three connections showed increased connectivity, linking right PreM&SMC with left DLPFC, FPA, and Broca’s area (Figure 4(B1,B2)).

#### 3.1.3. Comparison of Different Intervention Effects at the Channel Level

Baseline contrasts between the two sessions did not survive FDR correction (q < 0.05). In uncorrected comparisons, 48 channel pairs showed differences (uncorrected *p* < 0.05 and Cohen’s d ≥ 0.6), with 38 of these (79.2%) showing stronger baseline connectivity before the 1 Hz session and 10 (20.8%) showing stronger baseline connectivity before 10 Hz session (Figure 4(C1,C2)).

Direct post-stimulation comparisons also did not survive FDR correction at the edge level. Nevertheless, the uncorrected maps indicated a clear divergence between stimulation frequencies (Figure 4(D1,D2)). Among 207 channel pairs meeting the exploratory visualization criterion (uncorrected *p* < 0.05 and Cohen’s d ≥ 0.6), 204 (98.6%) showed stronger connectivity after 10 Hz than after 1 Hz stimulation. These differences were distributed widely across the brain, spanning both intra- and inter-hemispheric connections. Three connections showed the opposite pattern, with stronger connectivity after 1 Hz stimulation, involving left FPA with bilateral PreM&SMC and left OC with left Broca’s area.

To further evaluate whether these between-session differences reflected distinct within-session trajectories, we compared change scores (Δ = post − pre) between frequencies (Figure 4(E1,E2)). No Δ-based edge-wise effects survived FDR correction. In the uncorrected analysis, 276 channel pairs met the exploratory visualization criterion (uncorrected *p* < 0.05 and Cohen’s d ≥ 0.6). Of these, 275 (99.6%) showed a greater increase, or a smaller decrease, under 10 Hz than under 1 Hz, indicating a broadly more positive trajectory in the 10 Hz condition. Only one channel pair showed the opposite pattern, with a greater increase under 1 Hz, involving the left FPA with right PreM&SMC.

### 3.2. ROI-Level Functional Connectivity Networks

#### 3.2.1. Pre- vs. Post-10 Hz rTMS

**Figure 5:**
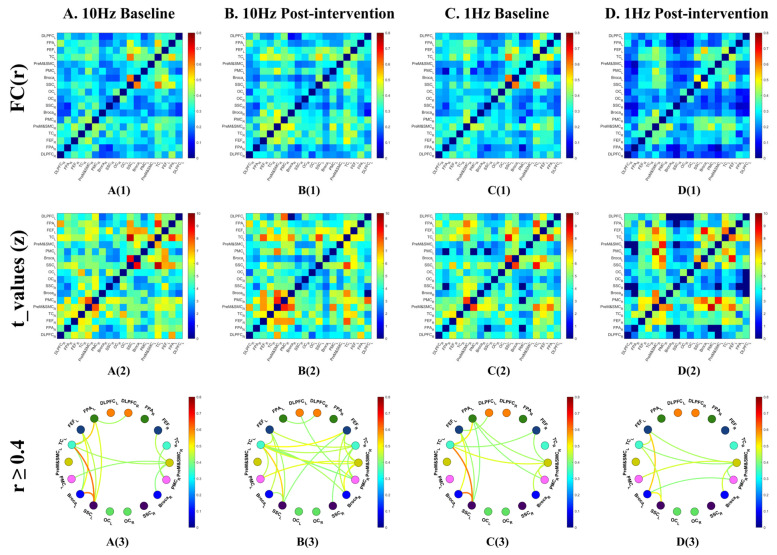
ROI-level functional connectivity architecture before and after 10 Hz and 1 Hz rTMS. (**A**) Baseline network before 10 Hz stimulation; (**B**) post-10 Hz network; (**C**) baseline network before 1 Hz stimulation; (**D**) post-1 Hz network. For each condition, panel (**1**) shows the full ROI-by-ROI functional connectivity (FC) matrix, panel (**2**) shows one-sample *t*-values for FC relative to zero (FDR-corrected q < 0.05), and panel (**3**) shows descriptively robust connections (r ≥ 0.4). The r ≥ 0.4 threshold was used for visualization only.

At baseline, all 153 ROI pairs showed significant functional connectivity relative to zero after FDR correction (q < 0.05) (Figure 5(A1,A2)). Among these, 15 connections met the descriptive threshold of r ≥ 0.4, primarily within left hemisphere frontal, temporal, and parietal regions (9 connections), with only two interhemispheric robust connections (Figure 5(A3)). After 10 Hz rTMS intervention, 151 ROI pairs remained significant relative to zero (q < 0.05, FDR corrected), and the number of robust connections increased to 24, with a notable increase in interhemispheric links (Figure 5(B1–B3)).

**Figure 6:**
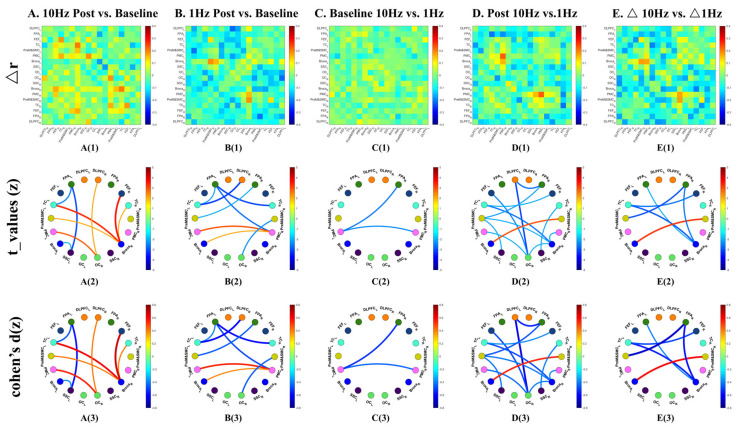
ROI-wise functional connectivity differences across rTMS frequency (10 Hz and 1 Hz) and time conditions (baseline and post-intervention). (**A**) Post-10 Hz versus baseline; (**B**) post-1 Hz versus baseline; (**C**) baseline 10 Hz versus baseline 1 Hz; (**D**) post-10 Hz versus post-1 Hz; (**E**) Δ10 Hz versus Δ1 Hz, where Δ = post − pre. Panel (**1**) shows FC difference values (Δr), panel (**2**) shows *t*-values corresponding to uncorrected *p* < 0.05, and panel (**3**) shows Cohen’s d for ROI pairs meeting the uncorrected *p* < 0.05 criterion. ROI-pair comparisons that did not survive FDR correction are presented as exploratory.

No ROI-pair pre–post differences survived FDR correction. In uncorrected analyses, nine ROI pairs showed notable changes (uncorrected *p* < 0.05; Cohen’s d = 0.40–0.67) (Figure 6(A1–A3)). Six of these connections showed increased connectivity, including links between right Broca’s area and FEF_R, TC_L, TC_R and PreM&SMC_L, as well as connections between DLPFC_R–OC_R and PMC_L–OC_R. Interestingly, three links showed decreased connectivity (d = 0.40–0.58), mainly involving the left FPA and left Broca’s areas with left SSC.

#### 3.2.2. Pre- vs. Post-1 Hz rTMS

The baseline ROI network before 1 Hz stimulation was similar to that before 10 Hz stimulation, with 149 ROI pairs significant relative to zero after FDR correction, and 15 robust connections (r ≥ 0.4) (Figure 5(C1–C3)). After 1 Hz stimulation, 140 ROI pairs remained significant (q < 0.05), while the number of robust connections decreased to 10 (r ≥ 0.4), with particularly reduced density in the left fronto-parieto-temporal network (Figure 5(D1–D3)).

No ROI-pair pre–post differences survived FDR correction. In uncorrected analysis, eight ROI pairs showed notable changes (uncorrected *p* < 0.05, d = 0.38–0.61). Six of these showed decreased connectivity (d = 0.41–0.61), involving FPA_L with TC_R and PMC_R; DLPFC_R with TC_L, FPA_R with PreM&SMC_L, and PreM&SMC_R with OC_R. Against this overall suppressive background, two connections were paradoxically strengthened, including connections between PMC_R and PMC_L (d = 0.54) and Broca_L (d = 0.38) (Figure 6(B1–B3)).

#### 3.2.3. Comparison of Different Intervention Effects at the ROI Level

Baseline differences between sessions did not survive FDR correction. In uncorrected comparisons, differences were limited and mainly involved PMC_L with FPA_R and PMC_R (uncorrected *p* < 0.05, d = 0.45–0.53) (Figure 6(C1–C3)).

Post-stimulation comparisons also did not yield ROI-pair effects surviving FDR correction. In uncorrected analyses, 13 ROI pairs showed differences (uncorrected *p* < 0.05, d = 0.39–0.64), and 12 of these (92.3%) were stronger after 10 Hz than after 1 Hz stimulation (d = 0.39–0.64), particularly among interhemispheric links (Figure 6(D1–D3)). The only ROI pair showing stronger connectivity after 1 Hz than after 10 Hz was Broca_L with PreM&SMC_R (d = 0.54).

A complementary change-score analysis (Δ = post − pre) was also performed at the ROI level (Figure 6(E1–E3)). No Δ-based ROI-pair effects survived FDR correction. In the uncorrected analysis, six ROI pairs showed divergent change trajectories between frequencies. Five of these (83.3%) showed a greater increase, or a smaller decrease, under 10 Hz than under 1 Hz. One ROI pair showed the opposite pattern, with a greater increase under 1 Hz than under 10 Hz, involving Broca_L with PreM&SMC_R.

### 3.3. Network-Level Topology and Spatial Summaries

**Figure 7:**
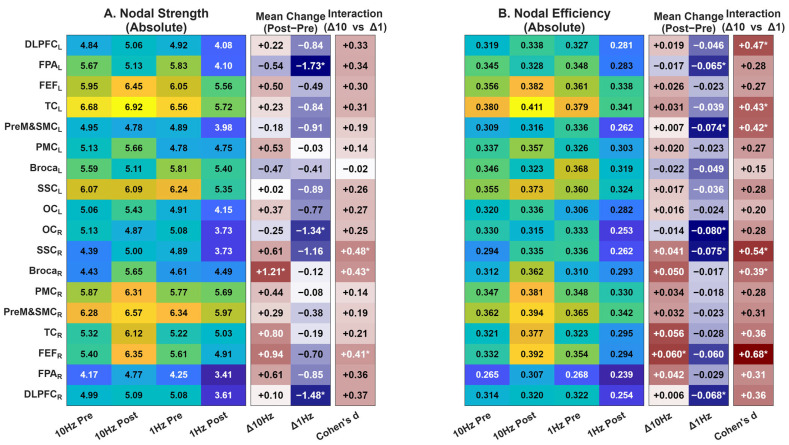
Node-wise topology summaries of frequency-dependent rTMS effects. Heatmaps show nodal strength (**A**) and nodal efficiency (**B**) across the 18 ROIs. In each panel, the left block displays the absolute mean values for the four experimental conditions (10 Hz pre, 10 Hz post, 1 Hz pre, and 1 Hz post). The middle columns show within-condition change scores (Δ10 Hz and Δ1 Hz, where Δ = post − pre). The rightmost column shows the between-frequency effect size for the change-score comparison (Δ10 Hz versus Δ1 Hz). These node-wise maps are presented as descriptive spatial summaries of topology-level effects rather than as primary inferential outcomes. Colors represent metric magnitude (blue to yellow) in the left panels, and directional changes (red for increase/positive, blue for decrease/negative) in the middle and right panels. * indicates *p* < 0.05.

At the network summary level, additional analyses of global topology, node-wise maps, and distance-stratified channel-level changes were performed (Table 1 and Table 2, Figure 7).

At the global topology level, repeated-measures ANOVA revealed significant Condition × Time interactions for global efficiency (F(1,29) = 4.473, *p* = 0.043, ηp^2^ = 0.134), local efficiency (F(1,29) = 7.098, *p* = 0.012, ηp^2^ = 0.197), clustering coefficient (F(1,29) = 6.140, *p* = 0.019, ηp^2^ = 0.175), and mean node strength (F(1,29) = 4.273, *p* = 0.048, ηp^2^ = 0.128) (Table 1). In each case, the interaction reflected relative stability or mild increases after 10 Hz stimulation, in contrast to decreases after 1 Hz stimulation. Small-worldness, modularity, and participation coefficient did not show significant interactions.

At the node level, the pattern was not spatially uniform (Figure 7). For nodal strength, larger between-frequency differences were observed in several right-hemispheric sensorimotor and frontal regions, including SSC_R, Broca_R, and FEF_R. For nodal efficiency, larger differences were observed in DLPFC_L, TC_L, PreM&SMC_L, SSC_R, and FEF_R, with the 1 Hz condition generally showing greater decreases than the 10 Hz condition.

To further summarize the channel-level changes by spatial scale, all channel-to-channel connections were grouped into four categories: intra-ROI (short-range), intrahemispheric (medium-range), interhemispheric homologous, and interhemispheric heterologous connections (Table 2). For each participant, mean change scores (Δ = Post − Pre) were computed within each category for each frequency, and Δ10 Hz versus Δ1 Hz was compared across participants. All four categories showed significantly more positive trajectories under 10 Hz than under 1 Hz, and these differences remained significant after Holm correction across the four categories. The largest difference was observed for intra-ROI short-range connections (t = −4.17, *p* < 0.001, d = −0.761), followed by intrahemispheric medium-range connections (t = −3.11, *p* = 0.004, d = −0.567), interhemispheric heterologous connections (t = −2.64, *p* = 0.013, d = −0.481), and interhemispheric homologous connections (t = −2.18, *p* = 0.038, d = −0.398).

## 4. Discussion

Using whole-brain fNIRS, this study examined the immediate effects of 10 Hz and 1 Hz rTMS over the left DLPFC on resting-state functional connectivity in healthy older adults. Across the dataset, the two stimulation frequencies showed different overall patterns of network reconfiguration: 10 Hz was generally associated with denser connectivity and relatively more positive network trajectories, whereas 1 Hz was associated with reduced connectivity and more negative trajectories. At the same time, only a small number of individual edge-wise effects survived stringent FDR correction. Accordingly, the broader connection-wise patterns are best viewed as exploratory, while the graph-theoretic metrics and distance-stratified summaries offer convergent network-level evidence for frequency-dependent differences in this sample.

Overall, the present findings are broadly consistent with our initial hypotheses, but they also suggest that the effects of rTMS in the aging brain are more complex than a simple “high-frequency excitatory, low-frequency inhibitory” account would imply. In particular, while 10 Hz tended to favor more integrated network organization and 1 Hz tended to favor functional sparsification, localized exceptions were observed in both directions. These paradoxical effects do not overturn the broader frequency-dependent pattern, but they do indicate that large-scale brain responses to rTMS are shaped by distributed network dynamics rather than by a purely local excitatory/inhibitory rule [13,18].

### 4.1. Validation of the Excitatory-Inhibitory Dichotomous Model

At the network summary level, the present data point to opposite large-scale effects of 10 Hz and 1 Hz stimulation. In the graph-theoretic analyses, stimulation frequency × time interaction effects were observed for global efficiency, local efficiency, clustering coefficient, and mean node strength, with 10 Hz showing relative stability or mild increases and 1 Hz showing decreases. The distance-stratified channel-level summaries showed the same directional pattern across short-, medium-, and long-range connection categories, and these category-level differences remained significant after Holm correction. Taken together, these results suggest that the divergence between frequencies was not limited to a few isolated edges, but extended across multiple spatial scales of the network.

Consistent with our first hypothesis, the observed global enhancement of whole-brain functional connectivity following 10 Hz rTMS is broadly consistent with excitatory effects at the network level. This enhancement was not confined to the stimulated left DLPFC but extended to numerous long-range and interhemispheric connections, suggesting that the impact of high-frequency rTMS may propagate through network cascades to influence distributed functional architecture. This interpretation is in line with literature suggesting that high-frequency rTMS can induce LTP-like plasticity and enhance the functional integration of large-scale brain networks [13,14,15,16].

At the mechanistic level, 10 Hz stimulation has been linked to NMDA receptor activation, calcium influx, and downstream CaMKII/CREB-related signaling, which may contribute to strengthening synaptic transmission efficiency [9,26,27]. The marked increase in interhemispheric connections observed here is also noteworthy and may be related to callosal transmission between homologous or functionally coupled regions [6]. Our data do not directly test these mechanisms, but they are compatible with the possibility that 10 Hz stimulation impacts not only local circuitry but also broader whole-brain coordination. In this sense, the emergence of more interhemispheric and longer-range connections may reflect greater communication across previously less connected functional modules. The connectivity enhancement centered on motor control networks (bilateral PMC, PreM&SMC) may likewise indicate increased integration of cognitive–motor control systems in the aging brain by 10 Hz rTMS.

In contrast, 1 Hz rTMS was associated with overall functional decoupling of the whole-brain network, accompanied by a marked decrease in the number of robust connections and a similar sparsification pattern at the ROI level. This reduction was widespread, involving both intrahemispheric and interhemispheric links. At the network summary level, the decreases in global efficiency, local efficiency, clustering coefficient, and mean node strength were all directionally consistent with this pattern. These findings are broadly consistent with our second hypothesis and with the classic “inhibitory” model of low-frequency stimulation, in which 1 Hz rTMS tends to reduce cortical excitability and promote a more segregated network state [13,14,15,16].

Mechanistically, 1 Hz stimulation has been linked to LTD-like processes, including phosphatase activation and AMPA receptor internalization, which may weaken synaptic strength [27,28]. If the neural activity of the left DLPFC is reduced, the driving influence of this region on downstream nodes may also weaken, with cascading effects across the broader network. In the present dataset, this was reflected not only in fewer robust connections but also in more negative change trajectories across short-, medium-, and long-range channel categories. Functionally, a certain degree of network segregation may reduce cross-network crosstalk and support more specific information processing [8,29,30]. However, in the present study this interpretation remains tentative, because no behavioral measures were collected to determine whether the observed reconfiguration was functionally beneficial, neutral, or maladaptive.

Direct comparisons between the two post-stimulation states showed that most observed differences favored stronger connectivity after 10 Hz than after 1 Hz stimulation. Likewise, the Δ-based comparisons showed overwhelmingly more positive trajectories under 10 Hz, with only a small number of exceptions. Taken together, these results suggest that the two frequencies were associated with opposite directions of large-scale network change, broadly consistent with the classical high-frequency excitation/low-frequency inhibition framework at the macro-network level [14,31]. At the same time, because most edge-wise effects did not survive multiple-comparison correction, this conclusion is better regarded as supported primarily by convergent descriptive and network summary evidence rather than by strong edge-wise confirmatory inference.

### 4.2. Beyond the Dichotomy: Explaining the Paradoxical Effects

Although the overall effects of the two stimulation frequencies diverged in the expected direction, the paradoxical connectivity changes observed in this study suggest that brain network modulation cannot be fully reduced to a simple excitation/inhibition dichotomy. In the present data, paradoxical effects included localized decreases following 10 Hz stimulation and localized increases following 1 Hz stimulation. These effects were not the dominant pattern, but they were sufficiently consistent across channel-level, ROI-level, and Δ-based analyses to merit consideration.

Connectivity reduction under 10 Hz stimulation: Against the background of global enhancement induced by 10 Hz stimulation, a minority of connections nevertheless showed reduced functional connectivity. For example, at the channel level, some frontoparietal connections and some links involving occipital and Broca-related channels weakened; at the ROI level, selected reductions involved left FPA with left SSC and TC. This paradoxical inhibitory effect is not fully captured by a single excitation model, and may reflect a more selective reorganization strategy of brain networks in response to external perturbation.

One possibility is that these reductions reflect competitive allocation and efficiency optimization of network resources. When 10 Hz rTMS preferentially strengthens certain core pathways, such as the motor-related networks highlighted here, the brain may downregulate connectivity in other non-critical or competing pathways in order to maintain efficient resource allocation [11,31,32,33]. Another possibility, particularly relevant to aging, is that some weakened links represent pruning or normalization of inefficient compensatory connections. Healthy aging is often accompanied by dedifferentiation, including reduced network specificity and increased between-network connectivity [21]. Under this view, the weakening of selected connections after 10 Hz stimulation may reflect a partial restoration of network specificity or functional compartmentalization, similar to observations in depression studies in which high-frequency rTMS has been associated with normalization of pathological hyperconnectivity [33,34].

A further possibility is that selective strengthening of core connections may lead to broader reorganization of network topology, potentially sharpening module boundaries rather than uniformly increasing all connections. In this sense, 10 Hz rTMS may not only enhance dialogue between nodes, but also reshape boundaries between subnetworks [7,35]. A network can, in principle, become more integrated overall while still showing greater selectivity in some between-network links, consistent with the idea that efficient brain organization depends on a balance between integration and segregation [36]. These interpretations are plausible, although the present data do not allow a definitive distinction between them.

Connectivity enhancement under 1 Hz stimulation: Against the background of global suppression induced by 1 Hz stimulation, a minority of connections showed the opposite pattern. At the channel level, three links were enhanced; while at the ROI level, the clearest examples involved bilateral PMC connectivity and connectivity between right PMC and left Broca’s area. Similar exceptions appeared in the Δ-based comparisons. This paradoxical facilitation effect has also been reported in previous studies [14,17,32,37], suggesting that the effects of low-frequency rTMS may involve more than simple inhibition.

One possible explanation is cortical disinhibition, potentially amplified by the physiological substrate of the aging brain [12]. The effects of rTMS depend not only on excitatory pyramidal neurons but also on inhibitory interneuron systems, particularly GABAergic circuits [38]. Under certain conditions, low-frequency stimulation may preferentially affect inhibitory interneurons, such as parvalbumin-expressing interneurons, thereby disinhibiting downstream pyramidal cells and increasing synchrony at the network level [38]. This mechanism may be especially relevant in older adults, because aging is accompanied by reduced inhibitory control and diminished GABAergic reserve [2,39,40,41]. In such a context, the inhibitory effect of 1 Hz stimulation on interneuron systems may more readily exceed the buffering capacity of the network, resulting in paradoxical enhancement of functional connectivity.

Homeostatic plasticity may also contribute to this pattern. According to the Bienenstock-Cooper-Munro theory, a reduction in cortical excitability can lower the threshold for subsequent strengthening, making enhancement effects more likely under certain conditions [19]. More broadly, homeostatic regulation helps maintain network stability by compensating for external perturbation [10,42]. In the aging brain, such compensation may overshoot, with selective increases in excitatory coupling emerging in response to inhibitory input. The enhanced bilateral motor cortex connectivity observed in this study may be compatible with such a compensatory response. When the cognitive control network centered on DLPFC is suppressed, the motor network may show relative strengthening to maintain a basic state of readiness, similar to delayed or compensatory effects reported in other distributed attention and sensorimotor systems [10,17,43].

At the same time, these mechanistic interpretations remain tentative. Some paradoxical effects may reflect visit-to-visit baseline variability or regression to the mean, especially in a crossover design with resting-state connectivity as the outcome. In addition, because the present analyses relied on HbO-based FC, residual systemic physiology or vascular responses may have contributed to apparent changes independent of neural coupling. This issue is particularly relevant in a TMS-fNIRS context, where stimulation may influence hemodynamics as well as neuronal activity. Therefore, the paradoxical enhancements observed here are best viewed as hypothesis-generating rather than as definitive evidence for a specific underlying mechanism.

### 4.3. Implications in the Context of Healthy Aging

The participants in this study were healthy older adults, and the findings should be interpreted in light of the characteristic neurobiological changes associated with aging. Older-adult brain networks commonly show weakened within-network specificity, increased between-network coupling, and reduced overall modularity [3,21]. In this context, the extensive emergence of interhemispheric connections after 10 Hz stimulation is compatible with the HAROLD model [4], in which bilateral recruitment serves as a compensatory strategy for maintaining function in older age. The relatively more positive trajectories observed after 10 Hz stimulation—particularly in the interhemispheric and longer-range summaries—may therefore reflect enhanced coordination in a system already relying on distributed compensation.

Meanwhile, these findings should not be interpreted as direct evidence that high-frequency stimulation counteracts aging-related network decline. A more cautious interpretation is that high-frequency stimulation may temporarily shift the aging network toward a more integrated state, which could be relevant to theories of compensation and maintenance. This interpretation is broadly in line with prior work suggesting that high-frequency rTMS can improve cognitive performance and modulate related connectivity patterns in older adults [7,12,44], but the present study did not include behavioral testing and therefore cannot establish functional benefit directly.

In contrast, the more negative trajectories following 1 Hz stimulation may indicate additional sparsification in a network that may already be vulnerable to reduced flexibility. This does not necessarily imply a uniformly maladaptive effect, but it does suggest that inhibitory protocols may have broader distributed consequences in older adults than would be predicted from a strictly local model. The paradoxical enhancement of motor cortex connectivity under 1 Hz stimulation is also noteworthy in this context. Because interhemispheric inhibition is already reduced in older adults [45], inhibitory stimulation of the left DLPFC may interact with an already altered inhibitory substrate, potentially facilitating compensatory increases in contralateral or bilateral motor coupling.

The motor-related findings deserve particular attention. Even against the background of overall suppression, motor-related links repeatedly showed selective strengthening under 1 Hz stimulation, whereas they also appeared prominently among the enhanced pathways after 10 Hz stimulation. This pattern may reflect a relative priority of the motor system in older adults, either because basic motor readiness is highly conserved or because motor networks are especially sensitive to rebalancing of frontal inhibitory control. Recent work has suggested that low-frequency stimulation can influence motor learning consolidation through modulation of inhibitory mechanisms [46], and the present results may be broadly compatible with that idea. Additionally, the decline in IHI function in older adults [45] may make the motor network more prone to compensatory enhancement. Still, because no younger comparison group was included, any age-specific interpretation remains indirect.

### 4.4. Potential Implications

The present findings may have implications for how whole-brain effects of rTMS are conceptualized in older adults. Rather than viewing stimulation only in terms of local excitability changes at the target, the current data support a broader network perspective in which stimulation is associated with distributed cortical reconfiguration. From this perspective, whole-brain fNIRS may be useful not only for describing whether connectivity increases or decreases after stimulation, but also for characterizing which spatial scales and network components are most sensitive to modulation, including interhemispheric, long-range, and motor-related pathways.

This network-level view may also be relevant to the development of more individualized neuromodulation strategies in aging populations. Older adults are likely to differ substantially in baseline network organization, compensatory patterns, and sensitivity to stimulation, and the present results suggest that the same nominal stimulation target can be associated with widespread but nonuniform effects across the brain. In this sense, whole-brain monitoring may help move the field away from a purely target-centered account of rTMS and toward a framework in which stimulation parameters are interpreted in relation to distributed network response. The paradoxical effects observed here further support this point, because they suggest that apparently counterintuitive changes may still reflect meaningful reorganization within a broader adaptive system.

However, the significance of these findings is primarily conceptual and methodological rather than immediately clinical. The present study did not include sham stimulation, behavioral outcomes, physiological monitoring, or longer-term follow-up, and therefore does not support direct claims regarding cognitive enhancement or clinical efficacy. A more appropriate interpretation is that the present work provides preliminary network-level evidence that may inform future sham-controlled, longitudinal, and behavior-linked studies of individualized neuromodulation in older adults. If such findings are replicated and linked to functional outcomes, whole-brain fNIRS may prove useful as a practical tool for protocol selection, response monitoring, and the refinement of network-informed stimulation strategies.

### 4.5. Limitations

This study has several limitations. First, the absence of statistically significant findings following stringent FDR correction at most individual edges likely reflects limited statistical power for high-dimensional connectivity inference with the present sample size (*n* = 30). Accordingly, the connection-wise results should be regarded as exploratory, even though the network summary analyses provided more stable support for frequency-dependent divergence. In addition, the stimulation dose used here (1200 pulses at 90% RMT) was relatively conservative. While this may be sufficient to induce transient changes [32,42], it may be insufficient to induce larger or more durable whole-brain reorganization, which often requires repeated or higher-dose stimulation in clinical settings [15]. This may be particularly relevant in a healthy older sample, where homeostatic mechanisms may counteract weaker external perturbations [13,15,36].

Second, the experimental design limits direct translational interpretation. The study did not include a sham stimulation condition, connectivity was assessed only immediately after stimulation, and no behavioral or cognitive outcome measures were collected. As a result, it remains difficult to separate stimulation-specific effects from nonspecific time or expectancy effects, to determine the temporal evolution, delayed emergence, or durability of the observed network changes, or to link those changes directly to functional outcomes.

Third, several methodological factors may also have influenced the results. The stimulation target was localized at F3, a practical and widely used scalp-based approximation of the left DLPFC when individual MRI guidance is unavailable, rather than with MRI-guided neuronavigation; this may have introduced additional targeting variability because scalp-based localization is less anatomically precise than image-guided targeting [47,48]. Moreover, the analyses relied on HbO-based resting-state connectivity without physiological monitoring or short-separation regression, leaving open the possibility of residual systemic or vascular influences on the estimated connectivity patterns. Although fNIRS is well suited to rTMS studies, its restricted penetration depth and lower spatial resolution mean that the present results are limited primarily to cortical surface networks. As a result, we are likely observing only part of the whole-brain effects induced by rTMS, as the present design cannot assess subcortical structures that are also important for large-scale network organization, such as the thalamus, basal ganglia, and hippocampus. Future studies incorporating multimodal imaging approaches, such as simultaneous EEG or fMRI, would help validate and extend the present findings.

Finally, the sample itself imposes constraints on interpretation. The study included only healthy older adults, and no younger comparison group was available; therefore, age-specific interpretations remain indirect. Education level was also not collected, limiting our ability to consider the possible influence of cognitive reserve on baseline network organization and responsiveness to stimulation.

Future studies should therefore include larger samples, sham control, longer follow-up intervals, behavioral outcomes, physiological monitoring, and, ideally, neuronavigation and younger comparison groups to more clearly define the robustness and functional significance of frequency-dependent rTMS effects.

## 5. Conclusions

This study suggests that 10 Hz and 1 Hz rTMS over the left DLPFC are associated with different patterns of immediate whole-brain network reconfiguration in healthy older adults. In general, 10 Hz stimulation was associated with relatively more positive changes in global topology and spatially distributed connectivity summaries, whereas 1 Hz stimulation showed the opposite overall trend. The observation of localized paradoxical effects further suggests that rTMS responses in the aging brain may involve more complex forms of reorganization than a simple excitatory/inhibitory dichotomy would predict. As an exploratory whole-brain study, the present work provides preliminary support for a network-level perspective on neuromodulation in older adults, while underscoring the need for larger, sham-controlled, behavior-linked, and longitudinal studies to determine the robustness and functional significance of these effects.

## Figures and Tables

**Table 1:** Global network and graph-theoretic topology metrics before and after 10 Hz and 1 Hz rTMS.

Network Metric	10 Hz Pre	10 Hz Post	1 Hz Pre	1 Hz Post	F (Cond × Time)	*p*	ηp^2^
Global Efficiency	0.365 ± 0.143	0.381 ± 0.147	0.369 ± 0.136	0.332 ± 0.098	4.473	0.043 *	0.134
Local Efficiency	0.330 ± 0.144	0.353 ± 0.154	0.337 ± 0.141	0.294 ± 0.102 ^†^	7.098	0.012 *	0.197
Clustering Coefficient	0.315 ± 0.151	0.337 ± 0.164	0.321 ± 0.150	0.277 ± 0.108 ^†^	6.140	0.019 *	0.175
Small-worldness (σ)	0.963 ± 0.186	0.980 ± 0.128	0.981 ± 0.213	0.914 ± 0.112	1.698	0.203	0.055
Modularity (Q)	0.119 ± 0.095	0.117 ± 0.098	0.114 ± 0.086	0.134 ± 0.082	1.001	0.325	0.033
Participation Coefficient	0.419 ± 0.106	0.415 ± 0.093	0.451 ± 0.097	0.434 ± 0.087	0.217	0.645	0.007
Mean Node Strength	5.329 ± 2.719	5.631 ± 2.933	5.380 ± 2.681	4.647 ± 1.896	4.273	0.048 *	0.128

Note. Values are mean ± SD. Condition × Time interaction effects were tested using repeated-measures ANOVA. Graph-theoretic metrics were computed from weighted ROI-level Fisher z-transformed connectivity matrices. Small-worldness (σ) was computed as (C/Crand)/(L/Lrand), where Crand and Lrand were estimated from degree- and density-matched random networks (100 realizations). * *p* < 0.05 for the Condition × Time interaction. ^†^ *p* < 0.05 for within-frequency pre–post paired comparisons. These analyses were used as network summary measures of frequency-dependent topology change.

**Table 2:** Distance-stratified summaries of channel-level connectivity change trajectories.

Connection Spatial Range	N_edges	10 Hz Δ (Post − Pre)	1 Hz Δ (Post − Pre)	t (Δ10 vs. Δ1)	*p*	Cohen’s d
Intra-ROI (Short)	270	+0.024	−0.071	−4.17	**<0.001**	−0.761
Intra-hemi (Med)	2180	+0.013	−0.062	−3.11	**0.004**	−0.567
Inter-hemi homolo (Long 1)	320	+0.004	−0.054	−2.18	**0.038**	−0.398
Inter-hemi heterolo (Long 2)	2180	+0.010	−0.055	−2.64	**0.013**	−0.481

Note. Channel-to-channel connections were classified into four spatial categories: intra-ROI (short-range), intrahemispheric (medium-range), interhemispheric homologous, and interhemispheric heterologous. For each participant and condition, participant-level mean change scores (Δz = post − pre) were computed within each category. Paired comparisons of Δ10 Hz versus Δ1 Hz were then performed across participants. *p* values shown here remained significant after Holm correction across the four spatial categories. N_edges indicates the number of valid channel-to-channel connections in each category. Bold values indicate statistical significance (*p* < 0.05).

## Data Availability

The datasets generated and/or analyzed during this study are available from the corresponding author upon reasonable request.

## References

[1] Damoiseaux J.S. (2017). Effects of aging on functional and structural brain connectivity. NeuroImage.

[2] Verstraelen S., Cuypers K., Maes C., Hehl M., Van Malderen S., Levin O., Mikkelsen M., Meesen R., Swinnen S.P. (2021). Neurophysiological modulations in the (pre)motor-motor network underlying age-related increases in reaction time and the role of GABA levels—A bimodal TMS-MRS study. NeuroImage.

[3] Grady C. (2012). The cognitive neuroscience of ageing. Nat. Rev. Neurosci..

[4] Cabeza R. (2002). Hemispheric asymmetry reduction in older adults: The HAROLD model. Psychol. Aging.

[5] Cabeza R., Albert M., Belleville S., Craik F.I.M., Duarte A., Grady C.L., Lindenberger U., Nyberg L., Park D.C., Reuter-Lorenz P.A. (2018). Maintenance, reserve and compensation: The cognitive neuroscience of healthy ageing. Nat. Rev. Neurosci..

[6] Liu Y., Hsu C.-C.H., Huang C.C., Zhang Y., Zhao J., Tsai S., Chen L., Lin C.-P., Lo C.Y.Z. (2021). Connectivity-Based Topographical Changes of the Corpus Callosum During Aging. Front. Aging Neurosci..

[7] Belov V., Erlandsson S., Magnusson M., Wårdell K., Zsigmond P. (2023). Subject-specific whole-brain parcellations of nodes and boundaries are modulated differently under 10 Hz rTMS. Sci. Rep..

[8] Finc K., Bonna K., Lewandowska M., Wolak T., Nikadon J., Dreszer J., Duch W., Kühn S. (2020). Dynamic reconfiguration of functional brain networks during working memory training. Nat. Commun..

[9] Huang Y.Z., Edwards M.J., Rounis E., Bhatia K.P., Rothwell J.C. (2005). Theta Burst Stimulation of the Human Motor Cortex. Neuron.

[10] Jargow J., Wilf M., Lavidor M. (2021). Low-Frequency TMS Results in Condition-Related Dynamic Activation Changes of Stimulated and Contralateral Inferior Parietal Lobule. Front. Hum. Neurosci..

[11] Singh A., Erwin-Grabner T., Sutcliffe G., Paulus W., Dechent P., Antal A., Goya-Maldonado R. (2019). Personalized repetitive transcranial magnetic stimulation temporarily alters default mode network in healthy subjects. Sci. Rep..

[12] Verstraelen S., van Dun K., Hehl M., Levin O., Meesen R., Swinnen S.P., Cuypers K. (2020). Induced Suppression of the Left Dorsolateral Prefrontal Cortex Favorably Changes Interhemispheric Communication During Bimanual Coordination in Older Adults–A Neuronavigated rTMS Study. Front. Aging Neurosci..

[13] Chervyakov A.V., Chernyavskaya P.O., Sinitsyn D.O., Piradov M.A. (2015). Possible Mechanisms Underlying the Therapeutic Effects of Transcranial Magnetic Stimulation. Front. Hum. Neurosci..

[14] Eldaief M.C., Halko M.A., Buckner R.L., Pascual-Leone A. (2011). Transcranial magnetic stimulation modulates the brain’s intrinsic activity in a frequency-dependent manner. Proc. Natl. Acad. Sci. USA.

[15] Fitzgerald P.B., Fountain S., Daskalakis Z.J. (2006). A comprehensive review of the effects of rTMS on motor cortical excitability and inhibition. Clin. Neurophysiol..

[16] Hanlon C.A., Dowdle L.T., Henderson J.S. (2018). Modulating Neural Circuits with Transcranial Magnetic Stimulation: Implications for Addiction Treatment Development. Pharmacol. Rev..

[17] Cocchi L., Sale M.V., Gollo L.L., Bell P.T., Nguyen V.T., Zalesky A., Breakspear M., Mattingley J.B. (2015). Dissociable effects of local inhibitory and excitatory theta-burst stimulation on large-scale brain dynamics. J. Neurophysiol..

[18] Hussain S.J., Freedberg M.V. (2025). Debunking the Myth of Excitatory and Inhibitory Repetitive Transcranial Magnetic Stimulation in Cognitive Neuroscience Research. J. Cogn. Neurosci..

[19] Siebner H.R., Lang N., Rizzo V., Nitsche M.A., Paulus W., Lemon R.N., Rothwell J.C. (2004). Preconditioning of Low-Frequency Repetitive Transcranial Magnetic Stimulation with Transcranial Direct Current Stimulation: Evidence for Homeostatic Plasticity in the Human Motor Cortex. J. Neurosci..

[20] Sporns O. (2013). Network attributes for segregation and integration in the human brain. Curr. Opin. Neurobiol..

[21] Ferreira L.K., Busatto G.F. (2016). Aging Effects on Whole-Brain Functional Connectivity in Adults Free of Cognitive and Psychiatric Disorders. Cereb. Cortex.

[22] Xia A.W.L., Han J., Wang Z., Chen R. (2025). Investigating the hemodynamic response to iTBS of the left DLPFC: A concurrent iTBS/fNIRS study. Brain Stimul..

[23] Curtin A., Tong S., Sun J., Wang J., Onaral B., Ayaz H. (2019). A Systematic Review of Integrated Functional Near-Infrared Spectroscopy (fNIRS) and Transcranial Magnetic Stimulation (TMS) Studies. Front. Neurosci..

[24] Huppert T.J., Diamond S.G., Franceschini M.A., Boas D.A. (2009). HomER: A review of time-series analysis methods for near-infrared spectroscopy of the brain. Appl. Opt..

[25] Brigadoi S., Ceccherini L., Cutini S., Scarpa F., Scatturin P., Selb J., Gagnon L., Boas D.A., Cooper R.J. (2014). Motion artifacts in functional near-infrared spectroscopy: A comparison of motion correction techniques applied to real cognitive data. NeuroImage.

[26] Chen D., Chen Y., Yan M., Li Y., Li S., Zhang X. (2025). High-frequency repetitive transcranial magnetic stimulation upregulates BDNF expression and promotes synaptogenesis in mouse models of Parkinson’s disease. Neuroscience.

[27] Sumi T., Harada K. (2020). Mechanism underlying hippocampal long-term potentiation and depression based on competition between endocytosis and exocytosis of AMPA receptors. Sci. Rep..

[28] Chen R., Classen J., Gerloff C., Celnik P., Wassermann E.M., Hallett M., Cohen L.G. (1997). Depression of motor cortex excitability by low-frequency transcranial magnetic stimulation. Neurology.

[29] Yuanjun X., Guan M., Wang Y., Xu Z., Liu R., Wang J., Zhang T., Li J., Wang H., Li J. (2024). Targeting auditory verbal hallucinations in schizophrenia: Effective connectivity changes induced by low-frequency rTMS. Transl. Psychiatry.

[30] Zhang L., Xing Y., Li C., Wang Y., Liu R., Wang J., Zhang T., Li J., Wang H., Li J. (2025). Low-frequency rTMS modulates small-world network properties in an AVH-related brain network in schizophrenia. Front. Psychiatry.

[31] Addicott M.A., Hong L.E., Peciña M., Sitaram R., Gollub R.L., Averill C.L., Furst A.J., Gueorguieva R., Pridgen B., Cutter G.R. (2019). Low- and High-Frequency Repetitive Transcranial Magnetic Stimulation Effects on Resting-State Functional Connectivity Between the Postcentral Gyrus and the Insula. Brain Connect..

[32] Beynel L., Deng L., Crowell C.A., Dannhauer M., Palmer H., Szabo C., Luber B., Lisanby S.H., Peterchev A.V. (2020). Effects of repetitive transcranial magnetic stimulation on resting-state connectivity: A systematic review. NeuroImage.

[33] Liston C., Chen A.C., Zebley B.D., Drysdale A.T., Gordon R., Leuchter B., Voss H.U., Casey B.J., Etkin A., Dubin M.J. (2014). Default Mode Network Mechanisms of Transcranial Magnetic Stimulation in Depression. Biol. Psychiatry.

[34] Schiena G., Maggioni E., Pozzoli S., Brambilla P. (2021). Connectivity changes in major depressive disorder after rTMS: A review of functional and structural connectivity data. Epidemiol. Psychiatr. Sci..

[35] Belov V., Erlandsson S., Magnusson M., Wårdell K., Zsigmond P. (2021). High frequency rTMS modulates functional connectivity nodes and boundaries differently in the human brain. bioRxiv.

[36] Rubinov M., Sporns O. (2010). Complex network measures of brain connectivity: Uses and interpretations. NeuroImage.

[37] Xie Y., Guan M., Wang Y., Xu Z., Liu R., Wang J., Zhang T., Li J., Wang H., Li J. (2024). Low-frequency rTMS induces modifications in cortical structural connectivity-functional connectivity coupling in schizophrenia patients with auditory verbal hallucinations. Hum. Brain Mapp..

[38] Moretti J., Rodger J., Brizuela D., O’Connor D., Rodger J. (2022). Low intensity repetitive transcranial magnetic stimulation modulates brain-wide functional connectivity to promote anti-correlated c-Fos expression. Sci. Rep..

[39] Heise K., Zimerman M., Hoppe J., Gerloff C., Wegscheider K., Hummel F.C. (2013). The Aging Motor System as a Model for Plastic Changes of GABA-Mediated Intracortical Inhibition and Their Behavioral Relevance. J. Neurosci..

[40] Hermans L., Levin O., Maes C., van Ruitenbeek P., Heise K., Edden R.A.E., Cuypers K. (2018). Brain GABA Levels Are Associated with Inhibitory Control Deficits in Older Adults. J. Neurosci..

[41] Zuppichini M.D., Mikkelsen M., Harris A.D., Edden R.A.E., Puts N.A. (2024). GABA levels decline with age: A longitudinal study. Imaging Neurosci..

[42] Anil S., Katsoulas A., Pleiter T., Bruggeman A., Wiersma D., van der Gaag M., Veling W., Goutier W. (2023). Repetitive transcranial magnetic stimulation (rTMS) triggers dose-dependent homeostatic rewiring in recurrent neuronal networks. bioRxiv.

[43] Battelli L., Grossman E.D., Plow E.B., Sattler C., Soto D. (2017). Local Immediate versus Long-Range Delayed Changes in Functional Connectivity Following rTMS on the Visual Attention Network. Brain Stimul..

[44] Liu M., Zhang H., Li B., Wang T., Li C., Liu Y., Wang Y., Wang L., Zhou Y. (2025). High-Frequency rTMS Improves Visual Working Memory in Patients With aMCI: A Cognitive Neural Mechanism Study. CNS Neurosci. Ther..

[45] Talelli P., Waddingham W., Ewas A., Rothwell J.C., Ward N.S. (2008). The effect of age on task-related modulation of interhemispheric balance. Exp. Brain Res..

[46] Egger S., Wenderoth N., Luft A.R., Taubert M. (2025). Repetitive Magnetic Stimuli Over the Motor Cortex Impair Consolidation of a Balance Task by Suppressing Up-Regulation of Intracortical Inhibition. Eur. J. Neurosci..

[47] Herwig U., Satrapi P., Schönfeldt-Lecuona C. (2003). Using the International 10-20 EEG System for Positioning of Transcranial Magnetic Stimulation. Brain Topogr..

[48] Mir-Moghtadaei A., Caballero R., Fried P., Fox M.D., Lee K., Giacobbe P., Daskalakis Z.J., Blumberger D.M., Downar J. (2015). Concordance Between BeamF3 and MRI-neuronavigated Target Sites for Repetitive Transcranial Magnetic Stimulation of the Left Dorsolateral Prefrontal Cortex. Brain Stimul..

